# Multicausal analysis on psychosocial and lifestyle factors among patients undergoing assisted reproductive therapy – with special regard to self-reported and objective measures of pre-treatment habitual physical activity

**DOI:** 10.1186/s12889-020-09522-7

**Published:** 2021-04-23

**Authors:** Viktória Prémusz, Alexandra Makai, Beatrix Perjés, Orsolya Máté, Márta Hock, Pongrác Ács, Miklós Koppán, József Bódis, Ákos Várnagy, Kinga Lampek

**Affiliations:** 1 Faculty of Health Sciences, University of Pécs, Vorosmarty u. 4, Pécs, 7621 Hungary; 2 MTA-PTE Human Reproduction Scientific Research Group, University of Pécs, Édesanyák u. 17, Pécs, H-7624 Hungary; 3 Department of Obstetrics and Gynaecology, Medical School, University of Pécs, Édesanyák u. 17, Pécs, H-7624 Hungary

**Keywords:** Physical activity, GPAQ, Accelerometer, Assisted reproduction, IVF, Outcomes, Psychosocial factors, Lifestyle, Pregnancy rate, Live birth

## Abstract

**Background:**

National, regional and global trends in prevalence of infertility indicate its public health importance, however it effects various life dimensions of individuals and couples as well. Lifestyle habits may counteract with these factors. The aim of the study was the multicausal analysis of psychosocial and lifestyle factors undergoing assisted reproductive therapy (ART) with special regard to pre-treatment habitual physical activity (PA).

**Methods:**

In a cross-sectional, observational cohort study on ART patients (*N* = 60, age 34.6 ± 5.2 years, BMI 24.2 ± 4.9 kg/m^2^) with follow up on outcome measures a detailed description was given on PA patterns (ActriGraph GT3X, GPAQ-H) and on general and infertility related distress (BDI-13, FPI).

**Results:**

Respondents reported normal mood state (BDI-13) but moderately high infertility-related distress (FPI) in Social- and very high distress in Sexual Concern. It was revealed that time spent with recreational PA (RPA) could counteract with infertility-related distress (Social Concern *R* = -0.378, *p* = 0.013; Relationship Concern *R* = -0.365, *p* = 0.019).

In the presence of clinical pregnancy GPAQ-H RPA MET was significantly higher (*p* = 0.048), in the non-pregnant group cumulative values and work-related PA were higher. Correlations could be found between RPA time and the number of oocytes (*R* = 0.315, *p* = 0.045), matured oocytes (*R* = 0.339, *p* = 0.030) and embryos (*R* = 0.294, *p* = 0.062) by women who reached at least 150 min RPA (GPAQ-H). Multivariate linear regression revealed that the number of oocytes was positively influenced by the GPAQ-H recreation MET (*R*^*2*^ = 0.367; F = 10.994, *p* = 0.004; B = 0.005, *p* = 0.004, B Constant = 4.604). Regarding the number of embryos (*R*^*2*^ = 0.757, *F* = 17.692, *p* < 0.001, B Constant = 1.342) positive relationship was found with GPAQ-H RPA MET (B = 0.004, *p* < 0.001) and negative with BMI (B = -0.167, *p* = 0.038). It was disclosed (*R*^*2*^ = 0.958, *F* = 408.479, *p* < 0.001) that higher Very Vigorous Activity (ActiGraph) was accompanied with higher hCG (B = 63.703, *p* ≤ 0.001). However, time spent with moderate PA (GPAQ-H) (B = 0.002, SE = 0.001, Wald = 3.944, *p* = 0.047, OR = 1.002) was significantly associated with live births.

**Conclusions:**

Amount of PA alone did not have a positive effect on outcome of ART. Type and intensity seemed to be more significant. Existing differences in response to infertility due to recreational PA suggest the importance of the development of a specific intervention. The robust overestimation of PA in self-reports highlights the need to improve physical literacy of women undergoing ART.

## Background

National, regional and global trends in prevalence of infertility indicate its public health importance [1–7]. Multiple definitions on infertility are used in parallel from demographic [1, 8] or epidemiological point of view [9], or it could even be considered as a disability [10]. The current study is based on the clinical definition which describes infertility as “a disease of the reproductive system defined by the failure to achieve a clinical pregnancy after 12 months or more of regular unprotected sexual intercourse” following the International Classification of Diseases (ICD-11) [11].

Infertility could specifically affect various life dimensions of individuals or couples, such as depression, anxiety, social isolation, sexual dysfunction, psychological and social distress (PSD), and poorer marital adjustment [12–15]. It has been hypothesized that depression and anxiety may substantially have a negative effect on female reproduction or assisted reproductive treatment (ART) due to hormonal, neuroendocrine, or immunologic functioning and lead to poor outcomes [16–20]. The relationship between psychosocial stress in relation to the success of IVF/ICSI is still under discussion [21, 22]. It was demonstrated that pre-treatment levels of perceived anxiety and depression were significantly related to treatment outcome in in vitro fertilization (IVF) [23, 24]. For this reason, it is necessary to explore the fertility related PSD.

Benefits of regular physical activity to maintain physical, mental and social health are not called into question [25–31]. Positive effects on women’s health are proven in different contexts [32–35] underlining its importance in pregnancy as well [36–39]. Depending on intensity or duration, certain studies disagree on health effects of exercise or even PA in relation to ART [40–43]. These PA studies primarily focus on outcomes of ART and less on the PSD aspects during the course of the therapy.

Therefore, the aim of the current research was to describe PA and PSD patterns and their combined effects on the course and outcomes of the treatment in ART patients using a multi-causal model.

## Methods

### Study design and sample

#### Study design

A cross-sectional, observational cohort study was conducted with consecutive sampling at the Assisted Reproduction Unit, Department of Obstetrics and Gynaecology, University of Pécs, Hungary. All female patients with both female and male factors of infertility who were indicated for fertility treatment (IVF/ICSI) were consecutively invited to participate in the study. Participants were recruited according to the date of the fertility consultation. Inclusion criterions were BMI ≥ 18 kg/m^2^ and ≤ 38 kg/m^2^, 18 to 40 years of age, having undergone not more than three unsuccessful cycles and no significant health risk relevant to the ART procedure and outcome (metabolic and vascular diseases including diabetes mellitus, metabolic syndrome, fatty liver diseases and atherosclerosis, severe endometriosis (stage III or IV) and/or adenomyosis). Participants were not diagnosed with any mental disorders and had no significant physical or mobility impairments.

**Fig. 1:**
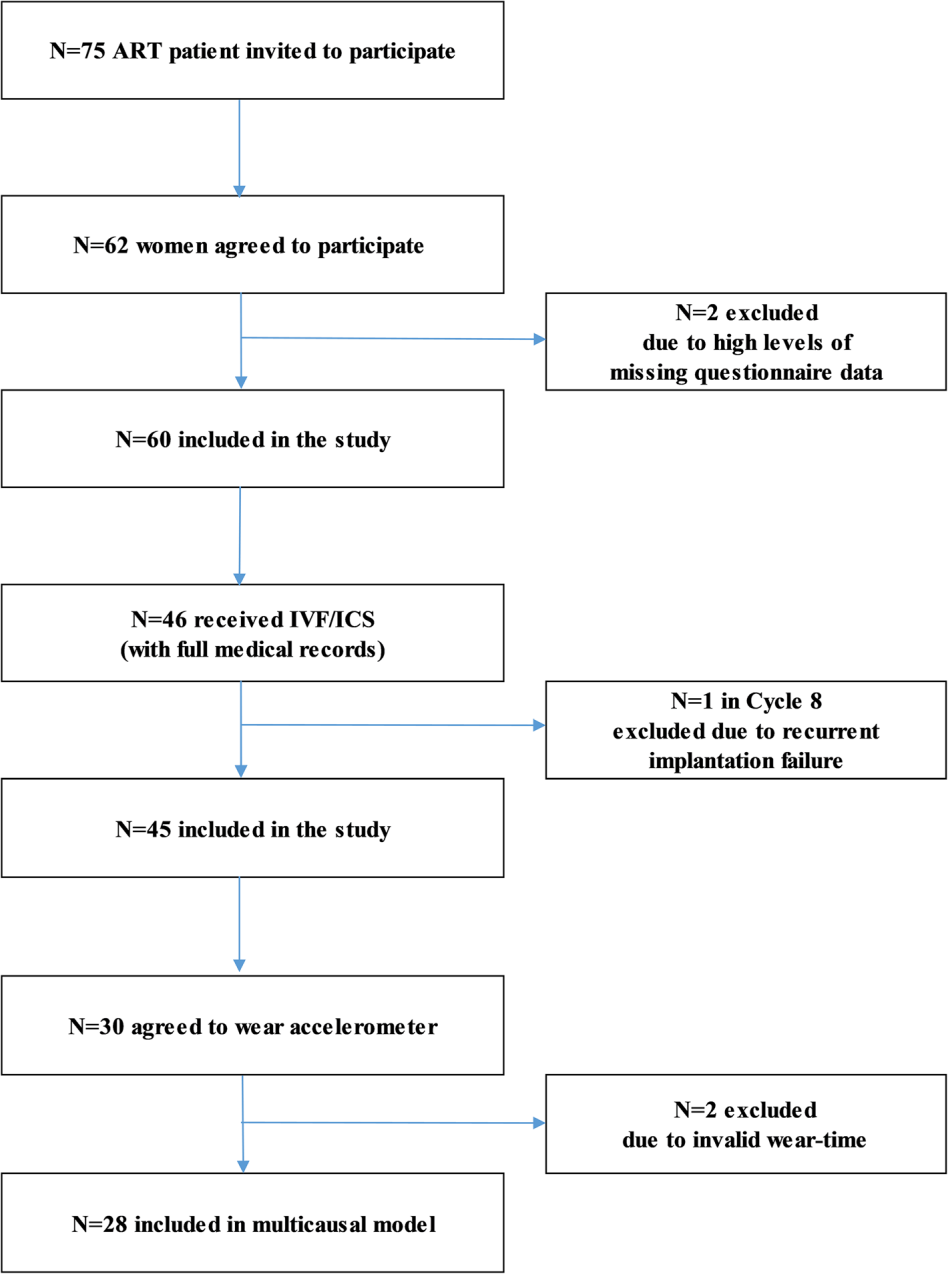
Flow chart of the study population selection including patient recruitment, exclusion criteria, and refusals

Data collection was carried out during the routine examination on the 3rd day of the unstimulated cycles. 62 women participated in the study between December 2018 and June 2019, which means 82.66% response rate. Self-administered questionnaires were given to participants, who filled them at home in a conventional paper-pencil form. Questionnaires were returned on the 21st day of the unstimulated cycles. 2 participants were excluded due to high rate of missing questionnaire data. The selection of the study population including patient recruitment, exclusion criteria, and refusals are presented on the flowchart (Fig. 1.)

#### General characteristics of the sample

The major socio-demographic and clinical characteristics of the study population have been presented in Table 1. The data of 60 female patients in reproductive age (34.6 ± 5.2 years), with mostly normal weight (70.0%, BMI 18.5–24.9 kg/m^2^) were analysed in the study. They were sampled from a larger proportion with higher educational degree (58.6%) and with satisfactory economic status (96.6%). 95.0% of them worked and 75.0% had urban residence. Each participant was either married or lived with a partner, and in average the duration of the partnership was 8 years (7.6 ± 3.8) with an around 5-years-long (59.0 ± 38.4 month) child-wish. We found various cases of infertility and types of treatments. However, these primarily nulliparous women (84.4%) typically received IVF/ICSI (82.3%) with mostly non-male indication (75.6%). Complete clinical data was available regarding 45 IVF/ICSI patients.

**Table 1:** General characteristics of women undergoing ART **(*N*** **= 60)**

Socio-demographic Data (***N*** = 60)		Health Status and Lifestyle (***N*** = 60)		Reproduction (***N*** = 60)		Medical records by IVF/ICSI (***N*** = 45)	
Age (years)		BMI (kg/m^2^)		***Indication (Self-report)***	N (%)	***Indication***	
Mean (SD)	34.6 (±5.2)	Mean (SD)	24.2 (±4.9)	Female	26 (43.3)	Poor semen quality	11 (24.4%)
***Education***		Underweight (< 18.5)	5 (8.3%)	Male	4 (6.7)	Fallopian tube	11 (24.4%)
2003Low	6 (10.0%)	Normal weight (18.5–24.9)	37 (61.7%)	Dual	14 (23.3)	Endometriosis	7 (15.6%)
Intermediate	18 (30.0%)	Overweight (25–29.9)	7 (11.7%)	Undefined	13 (21.7)	Other female	4 (8.9%)
High	36 (60.0%)	Obesity (> 30)	11 (18.3%)	Diagnosis in progress	3 (5.0)	Unexplained	12 (25.6%)
***Marital status (N = 44)***		***Self-Rated Physical Health***		***Type of ART Treatment***		***Procedures***	
Married	47 (78.3%)	Poor	0	IVF/ICSI	50 (82.3)	Cycle 1	11 (24.4%)
Partner	13 (21.7%)	Fair	1 (1.7%)	IUI	1 (2.1)	Cycle 2	16 (35.6%)
***Place of residence***		Neither good nor bad	13 (21.7%)	OI	2 (4.2)	Cycle 3	12 (26.7)
County seat	18 (30.0%)	Good	37 (61.7%)	HSG	2 (4.2)	Cycle 4	6 (13.3%)
City	27 (45.0%)	Excellent	9 (15.0%)	Examination in progress	5 (31.1)	Serum oestradiol - pmol/l	1692 ± 2073
Village	15 (25.0%)	***Healthy Diet***		***Child-wish (months)***	Mean ± SD	Progesterone - nmol/l	30.42 ± 21.29
***Income***		Pay attention	56 (93.3%)	Mean (SD)	59.0 ± 38.4	FSH^a^ - IU	2493 ± 2925
Low	2 (3.3%)	Not really / No attention	4 (6.7%)	***Relationship (years)***		Gonadotropin^a^ - IU	0.86 ± 0.19
Medium	34 (56.6%)	***Tobacco Use***		Mean (SD)	7.6 ± 3.8	No. of oocytes	7.87 ± 4.96
High	24 (40.0%)	Occasional	2 (3.3%)	***Gravidity (N = 45)***	N (%)	No. of matured oocytes^b^	5.44 ± 3.93
		Non-Smoker	58 (96.7%)	Nulligravid	25 (55.6)	No. of Grade 1 embryos	3.31 ± 2.98
		***Exercise***		Multigravid	20 (44.4)	No of transferred embryos	1.46 ± 0.84
		Regularly 4–7 days weekly	6 (10.0%)	***Parity(N = 45)***		hCG on day 12 - IU	364.9 ± 912.3
		Regularly1–3 days weekly	24 (40.0%)	Nulliparous	38 (84.4)	Chemical pregnancies	22 (48.89%)
		Not	30 (50.0)	Multiparous	7 (15.6)	Clinical pregnancies	13 (28.9%)

*ART* Assisted Reproductive Therapy, *BMI* Body Mass Index, *HSG* Hysterosalpingogram, *ICSI* Intracytoplasmic Sperm Injection, *IUI* Intrauterine Insemination, *IVF* In-Vitro Fertilization, *OI* Ovulation Induction, ^a^: Total dose administrated, ^b^ Metaphase II).

76.7% of the participants rated their physical health particularly good or excellent. In general, they self-reported a health-conscious lifestyle regarding diet, tobacco use and PA, and quality of sleep was satisfactory (86.2%) as well. Lifestyle change was also examined, but we cannot report relevant changes back to 5 years ago, since the beginning of ART, or in the current month. 50.0% of the participants claimed to be physically active, one tenth of them exercised 4–7 times per week (Table 1).

### Procedure and measurements

#### Assessment scales

Socio-demographic characteristics were obtained using questions regarding age, educational level, income, marital status, duration of partnership, duration of infertility, BMI and lifestyle habits.

#### Assessment of distress

Beck Depression Inventory (BDI-13) was applied for reporting respondents’ mental health status [23, 44–48]. The questionnaire represents how the subjects were feeling the week before. Each question has a set of four possible responses, ranging in intensity (0–3). A total score is computed reflecting the outcome index of depression. The validated Hungarian version of the short-form of the inventory with 13 items was completed by the respondents [49, 50].

To examine infertility-related distress with an infertility specific scale, the Fertility Problem Inventory (FPI) was included. FPI is a 46-item questionnaire developed to measure the level of infertility-related stress [51]. The scale consists of five subscales identifying the following domains: social concerns, sexual concerns, relationship concerns, rejection of childfree lifestyle and need for parenthood. Higher score indicates that the individual is experiencing more psychological stress than the average individual seen for infertility (85–98% reflects moderately high stress, and above 98% reflects very high level of stress). The former Hungarian version [52] was accurately redefined, results will be published separately.

#### Assessment of PA

To describe PA and exercise habits, participants self-reported on the type and frequency of exercise in a PA diary and reported all kinds of physical activity in GPAQ-H. These self-reports were compared with objective measures collected by the Triaxial ActiGraph GT3X+ accelerometers.

#### Global physical activity questionnaire (GPAQ-H)

The GPAQ version 2 was used in our research, which was developed by the WHO. This self-administered form comprises 16 items that measure the physical activity levels of a typical active week (7 days) of adults. The questionnaire contains three domains of PA: work, transportation, and recreational activities. The duration and frequency of physical activity (min/day) were recorded in case of all three above-mentioned domains. The evaluation of the intensity of certain activities is well introduced for the respondents in the questionnaires’ manual, based on the extent of increase in breathing or heart rate [53]. Results were expressed in time (minutes) or in energy expenditure (MET: Metabolic Equivalent of Task). According to intensity, moderate and vigorous activities can be classified and walking activities should be also distinguished.

Our study indicates data in min/week format for easier comparison with accelerometer data. Total MVPA min/week (all vigorous + all moderate activities’ mins), moderate and vigorous activities in min/week, and weekly sitting time in min/week values were calculated [53, 54].

The cultural adaptation, efficient translation, and validation of the Hungarian version were composed by our research group [54–56]. A total of 120 healthy adults (age 21.53 ± 1.75 years, 46.66% male) were included in the validity and reliability study, their last 7 days PA by GPAQ-H was compared with IPAQ-Hungarian Long version and Actigraph GT3X accelerometer data at the University of Pécs. Although, the validity GPAQ-H was fair to moderate (MVPA *R* = 0.290, *p* = 0.001) but it was acceptable, as by similar European studies. Consequently, it could be claimed that the GPAQ-H proved to be a valid and reliable questionnaire to measure the healthy Hungarian general population’s physical activity patterns [57].

For calculation of energy expenditure (MET) of PA following the guidelines of both of the questionnaires MPA by 4 MET, VPA by 8 MET and walking by 3.3 MET should be multiplied [53, 56, 58, 59]. We decided to apply the values of the updated “Physical Activity Guidelines for Americans”, which calculates with 3, 6 and 2.5 METs respectively [60].

#### Accelerometry monitoring

Triaxial ActiGraph GT3X+ accelerometers (ActiGraph, Pensacola, FL) were used to collect data on PA with standard device initialization (sample rate of 30 Hz, 60 s epochs and normal filter option).

Participants were instructed to wear the accelerometer on the right hip (near the iliac crest) for a week, from the time they woke up in the morning until they retreated at the end of the day, except for the duration of any water-based activities, such as swimming or bathing. The Actigraph GT3X + device measures the strength of the movement in three spatial directions, as well as their duration. The device converts acceleration into a quantifiable and measurable digital signal. It allows us to accurately assess daily activities and classifies it into categories.

Sixty or more motionless minutes were defined as “non-wear time”. A minimum of 480 min of wear-time was required daily and a minimum of 5–7 days with valid wear-time including at least one weekend day was required for inclusion in the analysis [61]. Finally, all valid days of recording were averaged and multiplied by seven to provide the comparability with the questionnaires.

ActiLife 6 software was used to initialize the accelerometer and to download results, and row data was converted with Freedson cut points [62]. The average of daily moderate to vigorous physical activity (MVPA) (min/day) and sedentary behaviour (SB) (min/day) was calculated [63], with a sensitivity and specificity of more than 98 and 99%, respectively [64].

Based on the additional physical activity diaries, there were no contact sports or water-based activities performed, which may restrain the participants to wear the accelerometer. However, four participants were excluded due to invalid wear-time. The average number of valid days was 6.32.

#### Physical activity categories by guidelines

Following the recommendation of the American Congress of Obstetricians and Gynaecologists (ACOG), pregnant women should engage in *moderate intensity exercise for 150 min* per week [65, 66]. However, there are no definitive physical activity guidelines for women attempting conception, or before or during assisted reproduction treatment.

To interpret our results, physical activity was categorized by meeting the key values of Physical Activity Guidelines for Americans for adults as *inactive* (any activity beyond basic movement from daily life activities), *insufficiently active (less than 150 min* of moderate-intensity physical activity (MPA) or 75 min of vigorous-intensity physical activity (VPA) or the equivalent combination of them per week), *active (equivalent of 150 min to 300 min* of MPA a week), or *highly active* (more than 300 min of MPA a week) [60]. Equivalent values were calculated through doubling by vigorous values and added to moderates.

PAQs scoring protocols are focusing on cumulative values of PA performed on average weeks or the week prior to the measurement. GPAQ categorises the level of physical activity as High, Moderate or Low by summing total PA [53], whereas the PA guidelines of PAGAC and ACOG focus on aerobe or exercise-type PA in relation to health enhancing effects. Therefore, we decided to analyse our data using the recreational type of activities following the PAGAC and ACOG categories [60, 65, 66].

#### Fertilization protocol

Publications of the Human Reproduction Scientific Research Group by Bódis and Várnagy described the detailed protocol of fertility treatments [67–69]. Patient enrolment into IVF procedure was approved by two independent physicians. The fertilization was performed with traditional IVF or intracytoplasmatic sperm injection (ICSI) depending on the andrological status (sperm count less than 15 M/ml), the maternal age (> 35) and the number of the previous IVF cycles the patient had before (> 2). Only metaphase II oocytes, identified by the presence of the first polar body, were chosen for fertilization. Embryo transfers were done 3–5 days after the oocyte retrieval. Only Grade 1 staged embryos were transferred, according to the Consensus embryo scoring system of ESHRE. To evaluate the success of the treatment, transvaginal ultrasound examination was performed 21 days after the embryo transfer to detect gestational sac [67].

One patient was excluded due to high risk of implantation failure, in her 8th cycle. The remaining patients took part in cycles 1–4.

### Statistical analysis

Statistical analyses were performed using IBM SPSS Statistics 25.0 for Mac (SPSS Inc., Chicago, IL, USA). Normality of data distribution was tested by Kolmogorov-Smirnov test. Mann-Whitney U-test was used to compare continuous variables. The association between two continuous variables was tested by Spearman’s rank correlation. To define predicting factors of primary and secondary outcomes of IVF from pre-treatment habitual PA, psycho-socio-demographic and baseline biomedical variables, we conducted a multivariate linear regression using the stepwise method. Logistic regression analysis was conducted to evaluate the effects of all the above parameters on live births. A post-hoc statistical power analysis was performed using G*Power software, version 3.1.9.6 for Mac (Franz Faul, Christian-Albrechts-Universität Kiel, Kiel, Germany) [70]. Data was expressed as mean ± SD as well as medians with 25th and 75th percentiles and the significance level of p<0.05 was considered in each case.

## Results

### General and infertility-related distress

The validated Hungarian short-form of the Beck Depression Inventory (BDI-13) was applied [44–47, 49, 50] to reflect on distress in general. 68.96% of the respondents scored less than 5 points, which indicates normal mood state; and 20.68% belonged to the category of mild depression (6–11 points). Two patients reported severe depression.

For the purpose of measuring the level of infertility-related stress, FPI was applied and moderately high *Global stress* (183.33 ± 28.19) was explored. In the five domains of the questionnaire we found similar values as in the pilot study [71]. Average stress by *Rejection of childfree lifestyle* (23.25 ± 6.04), moderately high stress by *Social concern* (41.40 ± 9.84) and very high stress level by *Sexual-* (38.62 ± 7.77) and by *Relationship concern* (48.53 ± 9.68). Stress related to *Need for parenthood* was low again, but markedly higher than in our first pilot study (31.68 ± 8.35 vs 23.1 ± 5.7). Table 2 shows these results.

**Table 2:** Pre-treatment distress characteristics of women undergoing ART (*N* = 45)

**BDI**					
	**Mean**	**SD**	**Median**	**IQR lower**	**IQR upper**
General Stress	4.92	4.82	4.00	1.00	8.00
**FPI**					
**Domains**	**Mean**	**SD**	**Median**	**IQR lower**	**IQR upper**
Social Concern	41.40	9.84	42.50	33.25	50.75
Sexual Concern	38.62	7.77	41.00	34.25	45.00
Relationship Concern	48.53	9.68	50.00	42.00	57.00
Rejection Concern	23.25	6.04	23.00	18.00	28.00
Need for Parenthood	31.68	8.35	31.00	28.00	37.00
Global Stress	183.33	28.19	179.50	165.00	202.50

### Descriptive analysis of physical activity patterns.

Pre-treatment physical activity patterns of women undergoing ART were summarised in Additional file 1. Intensity, frequency and mode of PA were described using the GPAQ-H questionnaire and ActiGraph GT3X accelerometer.

Regarding GPAQ-H, respondents performed an average of 461.50 ± 785.56 min/week moderate and 158.00 ± 467.34 min/week vigorous PA in work and only 35.00 ± 82.70 min/week vigorous activity in recreation/leisure time domain. However, medians (0.00) revealed that vigorous PA during work or leisure time are not common in the studied group. They preferred moderate intensity recreational activities for 2 h per week (124.80 ± 339.56).

Nevertheless, they spent 268.75 ± 521.77 min/week on average with active transportation, for example with walking or cycling, which covers 806.25 ± 1565.30 MET energy expenditure. Means significantly differed in these relations also, as only 120 min transportation was characteristic. They spent 6.53 h per day sedentary (2745.17 ± 1755.39 min/week).

Analysing the data by intensity, we found that respondents spent 786.32 ± 998.92 min (2910.65 ± 3932.02 MET) with moderate to vigorous activities (MVPA). In total, considering all types and intensities of activities lasting more than 10 min, women performed around 16.98 h (1018.95 ± 1225.72 min/week) or 3716.90 ± 4588.16 MET PA.

Regarding the ActiGraphs, light activity was the most typical with 1239.87 ± 329.50 min/week, moderate (233.35 ± 132.00 min/week) and vigorous activities (4.65 ± 13.27 min/week) lag behind the subjective measures, very vigorous activity was almost negligible (3.70 ± 15.73 min/week). They performed around 4 h MVPA (241.70 ± 145.10 min/week) and took in average 7060.28 steps daily (49,422.73 ± 16,351.52 counts/week) based on objective measures.

#### Comparative analysis of physical activity patterns

Comparing the data of the measurements, we found significant differences between the subjective instrument and the objective measures in all of the marked scores except for vigorous Accelerometer and GPAQ-H means (*p* = 0.255). (Table 3.)

**Table 3:** Comparison of pre-treatment physical activity characteristics of women undergoing IVF based on accelerometer, self-administered GPAQ-H questionnaires and ActiGraph GT3X mean values difference

Intensity	p	Mean Difference (min/week)
Sedentary	0.000	− 5980.21
Moderate	0.002	626.75
Vigorous	**0.255**	184.06
MVPA	0.001	805.23

To validate subjective PA results, we examined the correlation between accelerometer and questionnaires according to moderate, vigorous, MVPA activities, and sitting time values.

The GPAQ-H vigorous PA showed significant correlation with light accelerometer values (*R* = 0.310, *p* = 0.090). Time spent with transportation and the respective MET values showed moderate correlation with light activities (*R* = 0.506, *p* = 0.004) a tendency-like relationship (*R* = 0.349, *p* = 0.055) with objectively measured weekly steps, yet moderate negative correlation with sedentary time (*R* = -0.511, *p* = 0.003).

If we categorise their performance, 27 women (60.00%) reported notable leisure time PA, and only 18 of them (40.00%) reached the 150 min/week RMPA recommendation. 9 (20.00%) persons spent more than 240 min/week with recreational type PA, just like in the PAGA Highly active category. Comparison of pre-treatment physical activity characteristics of women undergoing IVF by physical activity categories is presented in Table 4.

**Table 4:** Comparison of pre-treatment physical activity characteristics of women undergoing IVF (*N* = 45) by physical activity categories

	Physical Activity Categories				
	Inactive	Insufficiently active	Active I	Highly active	Total
**Groups**	0 min/week	≤149 min/week	150–299 min/week	≥300 min/week	
**Non-pregnant**	14	6	4	8	32
	43.75%	18.75%	12.50%	25.00%	100.00%
	77.78%	66.67%	44.44%	88.89%	71.11%
**Pregnant**	4	3	5	1	13
	30.77	23.08	38.46	7.69	100.00%
	22.22%	33.33%	55.56%	11.11%	28.88%
**Total**	18	9	9	9	45
	40.00%	20.00%	20.00%	20.00%	100.00%
	100.00%	100.00%	100.00%	100.00%	100.00%

#### Relationship between psychosocial distress aspects and PA

Relationship between generic PSD, measured with BDI and PA patterns was not found. Our results on GPAQ-H revealed that recreational PA could counteract with some aspects of infertility related distress, since time spent with moderate RPA, total time and total MET of RPA negatively correlated with Social Concern (*R* = -0.378 *p* = 0.013, *R* = -0.386 *p* = 0.012 and *R* = -0.360 *p* = 0.023 respectively) and Relationship Concern of FPI (*R* = -0.365 *p* = 0.019, *R* = -0.368 *p* = 0.018 and *R* = -0.342 *p* = 0.033 respectively). However, time spent with vigorous RPA was also significantly correlated to ‘Rejection of childfree lifestyle’ (*R* = 0.354 *p* = 0.021). A relationship similar to the above cannot be described by ActiGarph.

#### Relationship between IVF outcomes and physical activity

If we divided IVF patients regarding the presence of clinical pregnancy, we can conclude that PA patterns differ. Due to high SD, significant difference was detected only in case of GPAQ-H recreational PA MET means (*p* = 0.048). Minutes spent with recreation per week also showed slight difference, but a level of significance was not reached (*p* = 0.067). In both cases, means of the pregnant group were higher.

If the tendencies were analysed by the subjective and objective measures, it could be seen that pregnant women spent more time and energy expenditure prior to the treatment with recreational type- or with vigorous activities, which refers to exercise. In contrast, in the non-pregnant group cumulative values of PA were higher, but in relation to work or in total, we assume that the amount of PA alone did not have a positive effect. Type and intensity of PA seems to be significant. (Additional file 2).

Consistent with the above results, if the pre-treatment PA measures undergoing IVF/ICSI were analysed by secondary outcomes, correlations can only be found with time spent with recreation. Significant relationship was found with the number of retrieved oocytes (*R* = 0.315, *p* = 0.045), number of matured oocytes (*R* = 0.339, *p* = 0.030) and slight trend with Grade 1 embryos (*R* = 0.294, *p* = 0.062) by women who reached at least 150 min RPA measured by GPAQ-H.

#### Multivariate linear regression analysis

To define predicting factors of primary and secondary outcomes of IVF from pre-treatment habitual PA, psycho-socio-demographic variables, and baseline biomedical variables, we conducted multivariate linear regression using the stepwise method.

We applied 3 models, which included women’s age, education, BMI, child-wish, duration of infertility and number of cycles, QoL and PSD parameters, and PA values as covariates. In the first step, we adjusted for age, education, and BMI. In the second step child-wish, duration of infertility and number of cycles were additionally adjusted. In the third step, we adjusted subscales of BDI and FPI as well, and finally, in the fourth step PA parameters as GPAQ-H and ActiGraph data were also included.

In Model 1 (*R*^*2*^ = 0.367) the number of oocytes, as the dependent variable was influenced positively by the GPAQ-H recreation MET (*F* = 10.994, *p* = 0.004; B = 0.005, *p* = 0.004, B Constant = 4.604).

The number of Grade 1 embryos was also examined as a dependent variable in Model 2 (*R*^*2*^ = 0.757, F = 17.692, *p* < 0.001, B Constant = 1.342). Positive significant relationship was found with GPAQ-H recreational physical activity MET (B = 0.004, *p* < 0.001) and negative relationship with BMI (B = − 0.167, *p* = 0.038).

When hCG levels on day 12 were considered as a dependent variable, multivariate linear regression disclosed in Model 3 (*R*^*2*^ = 0. 0.958, *F* = 408.479, *p* < 0.001) that higher Very Vigorous Activity level measured with ActiGraph was accompanied with higher hCG levels (B = 63.703, *p* ≤ 0.001).

#### Logistic regression analysis

On the basis of biomedical, psycho-socio-demographic and PA variables, logistic regression analysis was conducted to evaluate the effects of all the above parameters on live births. Contrary to our previous findings, the results indicated that total time (min/week) spent with moderate PA measured with GPAQ (beta coefficient [B] = 0.002, standard error [SE] = 0.001, Wald = 3.944, *p* = 0.047, OR = 1.002) significantly associated with live births.

## Discussion

It was assumed that the abundance of pre-treatment PA would decrease psychosocial distress domains in ART patients and thereby enhance reproductive performance. To assess the effects of psychosocial and lifestyle factors with special regard to physical activity on course and success of ART an observational cohort study was conducted with a follow-up of primary and secondary outcomes. To the best of our knowledge the current study was the first one in Hungary which gave a detailed description on the physical activity patterns of the specific cohort of patients undergoing assisted reproductive treatment using ActriGraph GT3X accelerometers and GPAQ-H questionnaire.

In general, 68.96% of the studied sample reported normal mood state (BDI-13), however they could be characterised by moderately high infertility-related distress (FPI), with moderately high level by Social- and very high level by Sexual concern.

Relationship between generic PSD, measured with BDI and PA patterns cannot be described. Our GPAQ-H results revealed that recreational PA could counteract with some aspects of infertility related distress, RPA negatively correlated with Social Concern and Relationship Concern of FPI. Significant difference cannot be described using PAGA PA categories or the 240 min cut off point regarding PSD.

Comparison of fertility-specific and general questionnaires can be found in literature in relation to ART patients [72–74]. Cserepes et al. conducted research using FertiQoL and Beck Depression Inventory on a Hungarian sample (126 couples). Female members of the couples reported poorer QoL than males. Subscales of the Core module scored between 69.01 ± 16.33 (Emotional Scale) and 80.26 ± 13.85 (Social scale), total QoL was described as 77.27 ± 12.05. These values were markedly higher than in our sample [75].

Domar et al. underline the role of improving mental health with psychological interventions in improved pregnancy rates among infertile women [76]. Other studies shift focus to lifestyle behaviours: Domar et al. made surprising observations regarding interfering health behaviours as exhausting exercise, smoking, regular consumption of alcohol and caffeinated beverages and taking herbal supplements during IVF cycles [77]. In our sample more health-conscious lifestyle could be observed.

The Fertility Experiences Project examined 202 first IVF cycle patients to predict the influence of psychological distress on IVF treatment outcome and subsequent PSD, in a prospective cohort study over an 18-month period using the Epidemiologic Studies Depression Scale (CES-D) and the State Anxiety Subscale of the State-Trait Anxiety Inventory (STAI). In a binary logistic model including covariates (woman’s age, ethnicity, income, education, parity, duration of infertility, and time interval), pre-treatment depression and anxiety were not significant predictors of the outcome measures. In linear regression models including covariates (woman’s age, income, education, parity, duration of infertility, assessment point, time since last treatment cycle, and pre-IVF depression or anxiety), experience of a failed IVF were associated with higher post-IVF depression and anxiety, which draw attention to the support of patients to prepare for and cope with treatment and treatment failure [78].

Regarding the examination of PA patterns, PAQs routinely overestimated all types and intensity of PA, but showed moderate correlation with objective values. Self-reported time spent sedentary was strongly correlated with questionnaires and accelerometer measures. Cumulative values of PA in average were analogous to the Hungarian general population, but medians demonstrated that most of these women completely avoided vigorous forms of PA and showed pre-treatment PA patterns like women during pregnancy.

Compared to the general Hungarian population, the subpopulation of the studied women cannot be described as inactive, however only 60.00% of the sample examined reported notable leisure time PA and only 40.00% reached the recommended level of 150 min/week recreational moderate physical activity. They spent 16.98 h per week with all forms of activity and spent 6.53 h per day sedentary. 50.00% of the women in the sample reported regular exercise, which could be considered as a relatively active subpopulation in Hungary compared to previous national studies [79, 80]. However, Ács et al. reported 10% improvement in PA habits based on representative Eurobarometer data from 2018: Hungarians’ regular sport participation and physical activity is 33%, which is below the EU average (40%). Authors noted that 42% of Hungarian citizens spent more than 2.5 and less than 5.5 h sedentary. With 6.53 h daily sitting time (2745.17 ± 1755.39 min/day), our results are slightly elevated but are in line with the above findings [81].

The amount of PA in our research increased unexpectedly in line with age and also with BMI, suggesting a more health-conscious behaviour by women exposed to higher risk. The current study did not include questions on health literacy, so we can only assume that these participants had some supposed knowledge on the relationship between reduced conception rate and overweight/obesity, insulin resistance or amount of visceral fat. Lungren, Kiel and co-authors developed a study protocol to provide knowledge on the results of a high-intensity interval training before ART in subfertile overweight or obese women and include the program in regular fertility care [82]. Similarly to other studies emphasizing the importance of PA on BMI [34], in a randomised controlled pilot trial authors proved, that the high-intensity interval training significantly improved insulin sensitivity, VO2 peak and abdominal fat. However, due to low number of participants (intervention group *N* = 8, control group *N* = 10) they could not draw a conclusion on pregnancy rate [82].

“Walking the way to better health” general recommendations promote 10,000 steps daily [83, 84]. With 7060.28 steps per day measured with accelerometers, our respondents cannot be categorised as inactive compared to a Nature letter by Althoff et al. on worldwide activity inequality, which mentioned the average daily steps to be around 5300 in Hungary, 5000 Worldwide and 4800 in the US, measured with smartphones [85]. In an update published in ACSM’s journal, Medicine & Science in Sports & Exercise authors revealed that 7000–9000 steps per day may trigger in health benefits, which are associated with current public health guidelines’ emphasis on minimal amounts of time spent in MVPA, the federally recommended amounts of 150 to 300 min per week [86].

Women were divided as per the abundance of clinical pregnancy. Pregnant women spent more time and energy expenditure with recreational type- or with vigorous activities, which refers to exercise. In contrast, in the non-pregnant group cumulative values of PA were higher, but in relation with work or in total, we assume that this alone did not have a positive effect. Type and intensity of PA seems to be significant. Significant relationship could be described with the number of retrieved oocytes, number of matured oocytes and sligth relationship with Grade 1 embryos by women who reached at least 150 min RPA measured by GPAQ-H.

It could be claimed that the GPAQ-H measurement tool is a valid and reliable questionnaire to measure the healthy Hungarian general population’s physical activity patterns [57]. By the validation study of GPAQ-H, Bland Altman plots revealed mean differences between the GPAQ-H and accelerometer data. The plots showed that GPAQ-H overestimates vigorous activities by 212.75 min/week (331.82–757.42) and MVPA values by 104.93 min/week (− 1016.98–807.11). In our current study we observed similar overestimation of vigorous activities with 331.10 vs 184.06 min/week, and considerable overestimation of MVPA 481.37 vs 805.23 min/week in case of GPAQ-H and IPAQ-SFH. In the validation study a high difference, 6336.79 min/week (CI 3638.18–9035.40) was revealed regarding sitting, as GPAQ-H largely underestimated the time spent sedentary [57].. Our study also revealed a difference in the measurement of sitting time between accelerometer and GPAQ-H − 5980.21 min/week.

Regarding GPAQ, our respondents performed moderate intensity PA during work and preferred that during recreation. However, mean values showed some vigorous activity in work (158.00 ± 467.34 min/week) and recreation (35.00 ± 82.70 min/week). Medians demonstrated that most of these women completely avoided vigorous forms of movement. Regarding female reproduction, there is a wide consensus on the beneficial effects of PA on gestation. Most studies draw attention to the risk of frequent vigorous PA on fertility [87, 88] and on success of ART [43, 77].

To describe PA levels, both instrumental and self-reported studies were published. Evenson et al. discussed that the adjusted odds of intrauterine gestation are higher among IVF patients who had higher continuous active living (OR 1.96, 95% CI 1.09–3.50), sports/exercise (OR 1.48, CI 1.02–2.15), and total activity (OR 1.52, 95% CI 1.15–2.01) indices in the past year [89].

Regarding the benefits of pre-treatment activity, Moran et al. reported positive effect of lifestyle intervention including exercise and diet in conjunction with ART in overweight and obese women and described elevated successful pregnancy rate (12 / 18 vs 8 / 20) in the intervention group compared to controls (Moran, Tsagareli, Norman, & Noakes, 2011).

Palomba et al. in their observational cohort study assessed the relationship between RPA and reproductive performance in connection with lifestyle interventions in obese infertile women who received ART (*N* = 216). Number of pregnancies (16/41, 39.0% versus 28/175, 16.0%, respectively; *p* = 0.002) and live births (10/41, 24.4% versus 13/175, 7.4%, respectively; *p* = 0.004) were significantly higher in 41 obese patients who did regular physical activity compared to 175 obese controls who did not. After adjusting for confounders, the relative risks for a clinical pregnancy and live birth were 3.22 (95% CI 1.53–6.78; *P* = 0.002) and 3.71 (95% CI 1.51–9.11; *P* = 0.004) in active patients, and RPA significantly correlated with improved reproductive performance irrespective of bodyweight loss [40]. In our study we could conclude significant difference between pregnant and non-pregnant groups by GPAQ-H recreational PA MET means (*p* = 0.048), which underline the importance of leisure time activities (inter alia) against PA in general.

On the other hand, adverse effects of excessive PA are also demonstrated. Gudmundsdottir et al. found that women who are active on most days, tended to experience fertility problems 3.2 times more often. In this study exercising to exhaustion also led to 2.3 times more fertility impairments than low intensity PA [90]. Based on the data by Morris et al. on lifetime exercise (level of evidence: II-2), exercising 4 h or more per week indicate 40% less likelihood of having a live birth (OR 0.6, CI 0.4–0.8), it is 3 times more likely to lead to cycle cancellation and 2 times more likely to lead to implantation failure or pregnancy loss (OR 2.8, CI 1.5–5.3; OR 2.0, CI 1.4–3.1; OR 2.0, CI 1.2–3.4 respectively) compared to non-exercise [43]. In the current research during the follow-up of IVF outcomes, particular attention was given to the women in our sample who reported at least 4 h exercise weekly (18.2%). In our study neither negative nor positive effects can be concluded by exceeding 240 or even 300 min of activity per week. Significant relationship could be described in relation to reproductive performance (number of retrieved oocytes and number of matured oocytes) by women who reached at least 150 min pre-treatment RPA measured by GPAQ-H.

Positive effects were also concluded in the meta-analysis of eight published studies (*N* = 3683 infertile couples) of Rao et al., which reported an increasing but not statistically significant trend in the implantation rate for physically active women when compared with physically inactive women (OR = 1.95, 95% CI 0.99–3.83, I2 = 77%). Rates of clinical pregnancy and live births in physically active women were significantly higher than those in physically inactive women (OR = 1.96, 95% CI 1.40, 2.73, I2 = 42% and OR = 1.95, 95% CI 1.06–3.59, I2 = 82%, respectively) [41].

Regarding PA immediately after IVF, Evenson et al. could not find any association between accelerometer-measured activity or sedentary behaviour with IVF outcomes. They described that after embryo transfer women engaged only in light activity (ME 3.0 h/day) and sedentary behaviours (ME 9.0 h/day). Although the current research focused on pre-treatment habitual PA, measurement of post-treatment PA in relation to QoL, PSD, and success rates could also offer research potential.

Espinós et al. reported in their meta-analyses based on 8 RCTs, that although lifestyle programmes improved pregnancy rates (RR: 1.43, CI: 95% 1.02 to 2.01; I2 = 60%; 8 RCTs; *N* = 1098), they had no impact on live births (RR: 1.39, CI: 95% 0.90 to 2.14; I2 = 64%; 7RCTs; *N* = 1034) and increased risk of miscarriage in obese infertile women [91]. In our sample, positive association was found between moderate PA and live births and no relationship with the ratio of miscarriage.

An analysis of 121,744 women with failed first treatment revealed that age is a key predictor of failure to have a live birth following IVF as well as the risk of hindered performance, while increased duration of infertility is also associated with poorer outcomes at every stage [92]. Comparing our results to our models, we cannot confirm the emphasized importance of PSD and age on reproductive performance.

To define predicting factors of primary and secondary outcomes of IVF from the point of view of PA and PSD, we conducted a multivariate linear regression using the stepwise method. We applied 3 models, which included women’s age, education, BMI, child-wish, duration of infertility and number of cycles and PSD parameters, and PA values as covariates. In Model 1 the number of oocytes was influenced positively by the GPAQ-H recreation MET, in Model 2 the number of Grade 1 embryos was positively correlated with GPAQ-H recreational physical activity MET and negatively with BMI. It was disclosed in Model 3 that higher Very Vigorous Activity level measured with ActiGraph was accompanied by higher hCG levels.

Gaskins and co-authors reported similar findings on maternal PA and sedentary behaviour in relation to ART’s reproductive outcomes. They found no association between MVPA time or total MET and outcomes as probability of implantation, clinical pregnancy or live birth. However, specific leisure time activities (aerobics, rowing, exercising with ski or stair machine) were positively associated with live birth (p-trend = 0.02).

### Limitations

The limitations of the study include the sample’s non-representative nature. To avoid potential confounders, patients were carefully selected, but made the study population modest. Objective measurement of PA patterns cannot be conducted by all patients and complete medical record was also missing in a portion of patients.

A post-hoc sample size estimation (using G*Power for Mac version 3.1.9.6) for the multivariate linear regression analysis (significance set at 5%, power set at 0.8, effects size at 0.15, and number of predictors at 2) showed that a total of 55 subjects would have been required to ensure adequate statistical power for analyses. The final sample of 45 subjects did not meet the sample requirements. Whereas the sample size was relatively suboptimal, given the limited study power, i.e. 71.84%, to detect the difference in primary and secondary outcomes of ART.

Attention was drawn by Gaskins et al. to the use of intermediate outcomes of IVF as surrogates of women’s reproductive performance [93]. Ongoing pregnancy has been considered as acceptable surrogate for live birth, as well as clinical pregnancy in or study. However, the major potential limitation of using ongoing pregnancies as the primary outcome of ART is the significant odds of pregnancy loss between the pregnancy confirmation and live birth.

For more impressive results on the effects of physical activity on the effectiveness of fertility programmes, a detailed objective assessment of physical activity, increased number of participants, and further examinations on outcome measures, with live birth’s rate as end point are needed in a well-powered randomized controlled prospective study.

## Conclusion

Infertility-specific scales could provide a more appropriate information on PDS of ART patients compared to general scales. GPAQ-H could be used as a valid measurement tool for mapping PA habits of ART patients, noting that the robust difference between objective and subjective measures (self-reports) of PA highlight the need to improve physical literacy of women undergoing ART.

Based on our results, recreation type of pre-treatment PA could positively influence domains of infertility related QoL and PSD during ART and improve reproductive performance in relation to primary and secondary outcomes. Existing differences in response to infertility due to PA suggest the need for the development of a specific intervention.

## Supplementary information

**Additional file 1 **Pre-treatment physical activity characteristics of women undergoing ART (*N* = 45) based on accelerometer, self-administered GPAQ-H questionnaire and ActiGraph GT3X data.

**Additional file 2 **Mean differences of pre-treatment physical activity measures undergoing IVF/ICSI (*N* = 45) by primary outcome.

## Data Availability

The dataset supporting the conclusions of this article is available from the corresponding author on reasonable request.

## Abbreviations

ACOG: American congress of obstetricians and gynaecologists
ART: Assisted reproductive therapy
BDI: Beck depression inventory
BMI: Body mass index
ESHRE: European society of human reproduction and embryology
FertiQol: Fertility quality of life tool
FPI: Fertility problem inventory
GPAQ-H: Global physical activity questionnaire-hungarian version
HADS: Hospital anxiety and depression
ICD-11: International classification of diseases 11th revision
ICSI: Intracytoplasmic sperm injection
IQR: Interquartile range
IUI: Intrauterine insemination
IVF: In-vitro fertilization
MVPA: Moderate to vigorous physical activity
MET: Metabolic equivalent of task
OI: Ovulation induction
PAQs: Physical activity questionnaires
RCT: Randomized controlled trial
