# Supplementary material for: Multicausal analysis on psychosocial and lifestyle factors among patients undergoing assisted reproductive therapy – with special regard to self-reported and objective measures of pre-treatment habitual physical activity
Source: BMC Public Health. 2021 Apr 23;21(Suppl 1):1480. doi: 10.1186/s12889-020-09522-7 (PMC8063288; doi:10.1186/s12889-020-09522-7)
Supplement: Supplementary file 1 — Additional file 1 Pre-treatment physical activity characteristics of women undergoing ART (N = 45) based on accelerometer, self-administered GPAQ-H questionnaire and ActiGraph GT3X data. [file 12889_2020_9522_MOESM1_ESM.docx]

#### Appendix 1.

#### Pre-treatment physical activity characteristics of women undergoing ART (N=45) based on accelerometer, self-administered GPAQ-H questionnaire and ActiGraph GT3X data

| **GPAQ-H** | | | | | | | | | | |
| --- | --- | --- | --- | --- | --- | --- | --- | --- | --- | --- |
| Measure | | Mean | | SD | | Median | | IQR lower | | IQR upper |
| **Work – VPA** | | | | | | | | | | |
| min/week | | 158.00 | | 467.34 | | **0.00** | | 0.00 | | 0.00 |
| MET | | 948.00 | | 2804.04 | | **0.00** | | 0.00 | | 0.00 |
| **Work – MPA** | | | | | | | | | | |
| min/week | | 461.50 | | 785.56 | | 75.00 | | 0.00 | | 630.00 |
| MET | | 1384.50 | | 2356.67 | | 225.00 | | 0.00 | | 1890.00 |
| **Transport** | | | | | | | | | | |
| min/week | | 268.75 | | 521.77 | | 112.50 | | 42.50 | | 232.50 |
| MET | | 806.25 | | 1565.30 | | 337.50 | | 127.50 | | 697.50 |
| **Recreation - VPA** | | | | | | | | | | |
| min/week | | 35.00 | | 82.70 | | **0.00** | | 0.00 | | 0.00 |
| MET | | 210.00 | | 496.19 | | **0.00** | | 0.00 | | 0.00 |
| **Recreation - MPA** | | | | | | | | | | |
| min/week | | 124.80 | | 339.56 | | 30.00 | | 0.00 | | 120.00 |
| MET | | 368.15 | | 1011.18 | | 90.00 | | 0.00 | | 360.00 |
| **Sitting** | | | | | | | | | | |
| min/week | | 2745.17 | | 1755.39 | | 2940.00 | | 1260.00 | | 3780.00 |
| **PA by intensity** | | | | | | | | | | |
| MPA min/week | | 594.12 | | 847.16 | | 240.00 | | 0.00 | | 720.00 |
| MPA MET | | 1752.65 | | 2530.33 | | 720.00 | | 0.00 | | 2122.50 |
| VPA min/week | | 193.00 | | 478.26 | | **0.00** | | 0.00 | | 120.00 |
| VPA MET | | 1158.00 | | 2869.56 | | **0.00** | | 0.00 | | 720.00 |
| MVPA min/week | | 786.32 | | 998.92 | | 300.00 | | 90.00 | | 1140.00 |
| **ActiGraph GT3X (N=30)** | | | | | | | | | | |
| Measure min/week | Mean | | SD | | Median | | IQR lower | | IQR upper | |
| Sedentary | 8598.43 | | 401.84 | | 8639.00 | | 8345.17 | | 8941.50 | |
| Light | 1239.87 | | 329.50 | | 1208.33 | | 1053.83 | | 1456.67 | |
| Moderate | 233.35 | | 132.00 | | 213.17 | | 134.36 | | 308.19 | |
| Vigorous | 4.65 | | 13.27 | | **1.00** | | 0.33 | | 1.75 | |
| Very Vigorous | 3.70 | | 15.73 | | **0.00** | | 0.00 | | 0.17 | |

GPAQ: Global Physical Activity Questionnaire, IQR: Interquartile Range, MET: Metabolic Equivalent of Task, min: minute, MVPA: Moderate to Vigorous Physical Activity, PA: Physical Activity
