# Supplementary material for: Multicausal analysis on psychosocial and lifestyle factors among patients undergoing assisted reproductive therapy – with special regard to self-reported and objective measures of pre-treatment habitual physical activity
Source: BMC Public Health. 2021 Apr 23;21(Suppl 1):1480. doi: 10.1186/s12889-020-09522-7 (PMC8063288; doi:10.1186/s12889-020-09522-7)
Supplement: Supplementary file 2 — Additional file 2 Mean differences of pre-treatment physical activity measures undergoing IVF/ICSI (N = 45) by primary outcome. [file 12889_2020_9522_MOESM2_ESM.docx]

#### *Appendix 2*

*Mean differences of pre-treatment physical activity measures undergoing IVF/ICSI (N=45) by primary outcome*

|  | | Non-pregnant | | Pregnant* | | Total | |  | |
| --- | --- | --- | --- | --- | --- | --- | --- | --- | --- |
|  |  | Mean | SD | Mean | SD | Mean | SD | Z | p |
| ActiGraph GT3X | Sedentary | **8294.74** | 1388.92 | 8135.17 | 2445.36 | 8265.73 | 1555.60 | -.341 | .733 |
|  | Light | **1092.32** | 396.67 | 1015.29 | 174.39 | 1078.32 | 364.21 | -.170 | .865 |
|  | Moderate | **211.65** | 139.16 | 199.79 | 130.87 | 209.49 | 134.71 | 0.000 | 1.000 |
|  | Vigorous | 1.24 | 1.66 | **5.17** | 9.68 | 1.95 | 4.24 | -.429 | .668 |
|  | Very Vigorous | 0.05 | 0.08 | **4.58** | 6.29 | 0.87 | 2.98 | -1.349 | .177 |
|  | Total MVPA | **212.94** | 140.51 | 209.54 | 140.15 | 212.32 | 137.08 | -.085 | .932 |
| GPAQ-H | WMPA m/w | **532.76** | 696.84 | 196.36 | 359.84 | 440.25 | 636.37 | -1.387 | .165 |
|  | WMPA MET | **1598.28** | 2090.53 | 589.09 | 1079.52 | 1320.75 | 1909.12 | -1.387 | .165 |
|  | WVPA m/w | **221.38** | 484.23 | 218.18 | 723.63 | 220.50 | 550.10 | -.889 | .374 |
|  | WVPA MET | **1328.28** | 2905.37 | 1309.09 | 4341.76 | 1323.00 | 3300.60 | -.889 | .374 |
|  | WPA m/w | **754.14** | 951.11 | 414.55 | 747.57 | 660.75 | 903.52 | -1.043 | .297 |
|  | WPA MET | **2926.55** | 4016.62 | 1898.18 | 4280.15 | 2643.75 | 4061.58 | -1.043 | .297 |
|  | RMPA m/w | 71.21 | 106.26 | **83.18** | 73.90 | 74.50 | 97.66 | -1.059 | .290 |
|  | RMPA MET | 213.62 | 318.79 | **249.55** | 221.70 | 223.50 | 292.97 | -1.059 | .290 |
|  | RVPA m/w | 25.86 | 54.81 | **68.18** | 104.00 | 37.50 | 72.77 | -1.059 | .289 |
|  | RVPA MET | 155.17 | 328.84 | **409.09** | 624.01 | 225.00 | 436.65 | -1.059 | .289 |
|  | RPA m/w | 97.07 | 129.67 | **151.36** | 108.26 | 112.00 | 125.22 | -1.834 | **.067** |
|  | RPA MET | 336.96 | 483.80 | **658.64** | 597.08 | 427.69 | 530.69 | -1.975 | **.048*** |
|  | TPA m/w | **299.14** | 523.72 | 184.09 | 227.67 | 267.50 | 461.43 | -.091 | .927 |
|  | TPA MET | **897.41** | 1571.16 | 552.27 | 683.01 | 802.50 | 1384.29 | -.091 | .927 |
|  | Sitting m/w | **3157.50** | 1678.52 | 2252.73 | 1794.46 | 2902.31 | 1737.64 | -1.598 | .110 |
|  | VPA m/w | 247.24 | 475.20 | **286.36** | 766.89 | 258.00 | 559.68 | -.370 | .711 |
|  | VPA MET | 1483.45 | 2851.19 | **1718.18** | 4601.36 | 1548.00 | 3358.05 | -.370 | .711 |
|  | MPA m/w | **603.97** | 738.65 | 279.55 | 350.13 | 514.75 | 666.83 | -.901 | .368 |
|  | MPA MET | **1811.90** | 2215.94 | 838.64 | 1050.40 | 1544.25 | 2000.50 | -.901 | .368 |
|  | MVPA m/w | **851.21** | 964.10 | 565.91 | 736.56 | 772.75 | 907.24 | -.486 | .627 |
|  | MVPA MET | **3295.34** | 3985.60 | 2556.82 | 4387.30 | 3092.25 | 4056.06 | -.395 | .693 |
|  | Total m/w | **1150.34** | 1230.55 | 750.00 | 728.43 | 1040.25 | 1120.71 | -.409 | .683 |
|  | Total MET | **4192.76** | 4828.50 | 3109.09 | 4300.56 | 3894.75 | 4660.57 | -.303 | .762 |
